# Health Status of North African Adolescent and Young Adult Migrants in Europe: a Scoping Review

**DOI:** 10.1007/s10903-025-01821-8

**Published:** 2025-11-15

**Authors:** Eva P. Rocillo Aréchaga, Barbara Broers, Catherine Chamay Weber, Delphine Courvoisier, Lloyd Orphée Rigumye, Melanie Pinon, Yves-Laurent Jackson

**Affiliations:** 1 Division of Primary Care Medicine, Geneva University Hospitals (HUG), Geneva, Switzerland; 2 Institute of Global Health, Faculty of Medicine, University of Geneva (UNIGE), Geneva, Switzerland; 3 Department of Community Health and Medicine, Faculty of Medicine, University of Geneva (UNIGE), Geneva, Switzerland; 4 Adolescent Health Unit, Division of General Paediatrics, Department of Women, Child and Adolescent, Geneva University Hospitals (HUG), Geneva, Switzerland; 5 Division of Quality of Care, Geneva University Hospitals (HUG), Geneva, Switzerland; 6 Department of Medicine, Faculty of Medicine, University of Geneva (UNIGE), Geneva, Switzerland; 7 University of Applied Sciences and Arts Western Switzerland, School of Health Sciences (HES-SO), Geneva, Switzerland

**Keywords:** Migrants, North Africa, Adolescents, Young adults, Health status, Scoping review

## Abstract

More than five million people from North Africa (NA) lived in Europe in 2019, including youth characterized by intense trans-border mobility, precarious living conditions and high-risk behaviours such as substance use, prostitution and illegal activities. This young population challenges health professionals and health service's ability to deliver medical care. The health literature about them remains scarce. This scoping review aims to map the existing knowledge regarding the health status of adolescent and young adult migrants from NA in Europe, the health services available to address their needs, and their access and use of these services. This scoping review followed the Arksey and O´Malley process and was based on the Population-Concept-Context framework. Six databases (CINAHL, Cochrane, Embase, PubMed, PsycINFO and Web of Science) were consulted for peer-reviewed articles and non-indexed reports. References of selected articles were systematically tracked. Initial search returned 5540 articles, 13 of which met the criteria for final inclusion. They originated from Spain, France and Germany. All records included were observational. Nine included only males and ten focused on unaccompanied minors. All studies explored aspects related to the health status of participants. Five explored aspects related to general health, seven to infectious diseases, six to mental health including substance use, four to oral health, and three to nutritional status. Two studies explored aspects related to the provision of care and related cultural elements. Evidence about the health status, access to services and health service provision of North African adolescent and young adult migrants in Europe is very limited, including important gaps about females and young adults, as well as about healthcare programs and use of services. Further research is needed to enhance a comprehensive, complex and reflective understanding of this population. Research design should emphasize gender perspective, include methodologies adapted to hard-to-reach populations and grounded into international collaboration.

**Supplementary Information:**

The online version contains supplementary material available at https://doi.org/10.1007/s10903-025-01821-8.

## Introduction

In 2022, more than 24 million people living in the European Union (EU) were migrant born outside EU borders [1], of whom a large number were born in North Africa (NA). As of mid-2019, there were an estimated 2.8 million Moroccans, 1.8 million Algerians, and more than 600,000 Tunisians living in European countries [2].

From the early 2000’s, immigration offices, Non-Governmental Organizations (NGOs), health professionals, and youth protection services in Europe reported an increasing number of young people migrating from NA countries in the context of booming and highly perilous non-regular transmediterranean transportation networks [3]. Most were young adults and adolescents, but some were as young as 10 to 12 years old, and usually travelling unaccompanied. Field observations and scientific studies described a sharp gender imbalance with a large majority of males, intense transborder mobility within EU countries, the frequent exposure to very precarious living conditions in absence of legal residence status, and regular interactions with the judiciary and penitentiary systems [3, 4]. The same sources pointed out specific health challenges and high-risk behaviours, to an extent not previously reported in other young migrant populations [3, 5]. The term “harragas” (from the word “harag” in Arabic, which means the “burners”) has been used in general and scientific literature to refer to them as those who burn their legal documents to avoid being identified and deported [6]. These adolescents and young adults were initially reported in Spain, and from 2010 in large cities of Italy, Belgium, Germany, Sweden, The Netherlands, Denmark, Finland and more recently Switzerland [3].

The main health needs reported in this group concerned physical and mental conditions, often associated with the use of psychotropic substances [3, 7]. Risky behaviours commonly observed include substance use, prostitution and illegal activities. Available literature in other groups indicates that these behaviours are associated with poor mental and physical health, premature mortality [8–11], as well as with multiple barriers to access health care services [12, 13]. The emergence of this population represented a new challenge for professionals delivering care to migrants compounded by specific social, legal and cultural aspects. Existing health services and practices do not seem to match their needs and demands, and professionals in all countries report important difficulties in delivering value-based care, both in terms of quality and continuity [14]. This may perpetuate and at least partly explain the poor health outcomes that are observed.

The health literature on migrants from NA in Europe is scarce. Studies focusing on adolescent and minor age groups are even rarer. Most existing studies explored domains related to social sciences and legal aspects, notably around the phenomena of unaccompanied minors. An initial literature search conducted in 2022 supported the hypothesis that the existing literature on their health status (physical and mental), use and appropriateness of health services delivery to them was limited and fragmented. This initial search revealed also that there is currently no systematic or scoping review of evidence on this topic. These knowledge gaps hinder the development and implementation of targeted health policies.

The objective of this scoping review is to map the existing knowledge regarding the health status of adolescent and young adult migrants from NA in Europe, the health services available to address their health needs, and their access and use of these services. Research questions guiding this scoping review were: (1) What is known about the health status of adolescent and young adult migrants from NA in Europe; (2) What is known about the clinical services in Europe to address the health needs of adolescent and young adult migrants from NA; and (3) What is known about the access to and use of healthcare services by adolescent and young adult migrants from NA in Europe.

## Methods

In the absence of a well-documented and structured body of knowledge on this topic we chose to conduct a scoping review to assess up to date relevant existing evidence from different sources [15]. A protocol was designed using the five steps process proposed by Arksey and O´Malley and refined in line with the recommendations made by the Joanna Briggs Institute (JBI) [16, 17]. Ethical clearance was not requested as this was a review of published literature.

### Inclusion and Exclusion Criteria

The search strategy was defined based on Population-Concept-Context (PCC) framework [18]. In this study, the population was composed of adolescent and young adult migrants from NA living in Europe. Migrants were defined as foreign-born people who moved to another country for the purpose of settlement, therefore including economic migrants, temporary foreign workers, foreign students, documented and undocumented migrants, and asylum seekers. Only first-generation migrants born in Algeria, Morocco, Tunisia, or Libya were included in this review. Male and female aged 10 to 24 years old were included in the study. Age was either self-reported or legally recognized by competent authorities.

Regarding concepts, this review included scientific reports and articles reporting about health status, health services, access and use of health services. Health status was determined as the level of health of the individual, group, or population either subjectively assessed by the individual or by objective indicators. It included physical and mental health, the latter being defined as those related to psychiatric, psychological and substance use conditions. We defined health services as all services provided for the diagnosis and treatment of a disease or injury and the maintenance of health, including harm reduction interventions, by any health professional. In addition, reports focusing on the ability of individuals to gain entry and to receive care and health services (or not) were included. Lastly, we included studies exploring the patterns of use of health services by the targeted population and those describing health behaviours.

The context was defined by the geographical area where the studies were conducted including the 53 countries of the WHO European region and all the types of health care settings (community, primary, secondary, and tertiary level) [19].

We included all reports published between 1990 and 2022. The year 1990 was chosen as it marks the beginning of public attention in Europe to this type of migration. Publications in English, French and Spanish in peer-reviewed journals and non-indexed reports were included in our search algorithms.

The detailed list of inclusion and exclusion criteria is presented in the Table 1.

**Table 1:** Studies inclusion and exclusion criteria

Field	Inclusion criteria	Exclusion criteria
Population	Population with all the following criteria • Migrants from North African countries, which include Morocco, Algeria, Tunisia, and Libya • First generation migrants, asylum seekers and undocumented migrants • Individuals from 10 to 24 years old	Studies reporting on adolescent and young migrants from NA together with individuals from different age groups, and not presenting results for the 10–24 age group
	Any gender	Studies reporting on migrants from NA, together with individuals from other origins and not presenting data for migrants from NA
Concept	Studies reporting at least one of the following concepts: • health status • health services • accessibility to health services • use of health services	
Context	Studies conducted in WHO European region (53 countries) *	Studies conducted outside the WHO European region
Type of study	Peer reviewed studies and non-indexed reports	Publications non-published as peer-reviewed or non-indexed reports, such as editorial letters, abstracts, books, book chapters, and thesis dissertations
Language	Publications in English, French or Spanish	
Year of publication	Reports published between 1990 and 2022	

*These include: Albania, Andorra, Armenia, Austria, Azerbaijan, Belarus, Belgium, Bosnia and Herzegovina, Bulgaria, Croatia, Cyprus, Czechia, Denmark, Estonia, Finland, France, Georgia, Germany, Greece, Hungary, Iceland, Ireland, Israel, Italy, Kazakhstan, Latvia, Lithuania, Luxembourg, Malta, Monaco, Montenegro, Netherlands, North Macedonia, Norway, Poland, Portugal, Republic of Moldova, Romania, Russian Federation, San Marino, Serbia, Slovakia, Slovenia, Spain, Sweden, Switzerland, Tajikistan, Turkey, Turkmenistan, Ukraine, United Kingdom of Great Britain and Northern Ireland, Uzbekistan

### Sources of Evidence

In line with JBI recommendations, we followed a three steps search strategy. The first was a search in the PubMed and EMBASE databases for entries relevant to the topic [20, 21]. Then, we conducted an analysis of the text words contained in the title and abstract of retrieved papers, and of the index terms used to describe the abstracts. In the second step, we translated all the identified keywords and index terms from this initial search in the following databases to be as comprehensive as possible: Cumulative Index to Nursing and Allied Health (CINAHL), Cochrane, Embase, PubMed, PsycINFO, and Web of science [20–25]. The search strategy was designed with the support of a team of medical librarians. (Annex 1). In the third step, we contacted the authors of all the identified records which were available only as abstracts to obtain full text documents. For all the identified records which were reviews, we searched for full text of original studies included. Finally, we consulted the reference list of all the selected articles to identify additional potential entries.

### Study Selection

Search results from each database were imported into EndNote 20 [26] and duplicates were removed. Two independent reviewers (ERA and YJ) screened the titles and abstracts of all entries and then conducted full text review of those potentially responding to the inclusion criteria. Discrepancies were resolved by a third reviewer (BB). To ensure reliability between reviewers, a series of three pilot tests were conducted before title and abstract screening. During each pilot test, 20 articles were used to evaluate the agreement between reviewers. The inclusion and exclusion criteria were discussed as a result of these pilot-test. Once agreement reached 70% concordance, we proceeded to the screening of all title and abstracts.

### Charting of Findings

Data was extracted using a descriptive charting table. This table was pretested before starting the data extraction (Annex 2). The following key information were extracted: Author(s); Year of publication; Publication type; Year of data collection; Country (ies) of intervention; Objective of the study; Study design and analysis method; Population characteristic (country of origin, age, sex, inclusion and exclusion criteria); Number of participants; Comparison group (if any); Concept described (e.g., health status, health service, accessibility and use of healthcare); Context (e.g., type of healthcare setting); Health status described; Healthcare service described; Provider characteristic; Accessibility issue described; Pattern of use identified; Outcomes, recommendations, and lessons learned; and any other topic relevant to our research question.

### Collating, Analysing and Presenting the Results

Findings are reported in line with Preferred Reporting Systematic Reviews and Meta-Analysis Scoping Review (PRISMA-ScR) [27]. Results are organized by studied population, health status (including by each main category), and health services.

## Results

### Searching Results

**Fig. 1:**
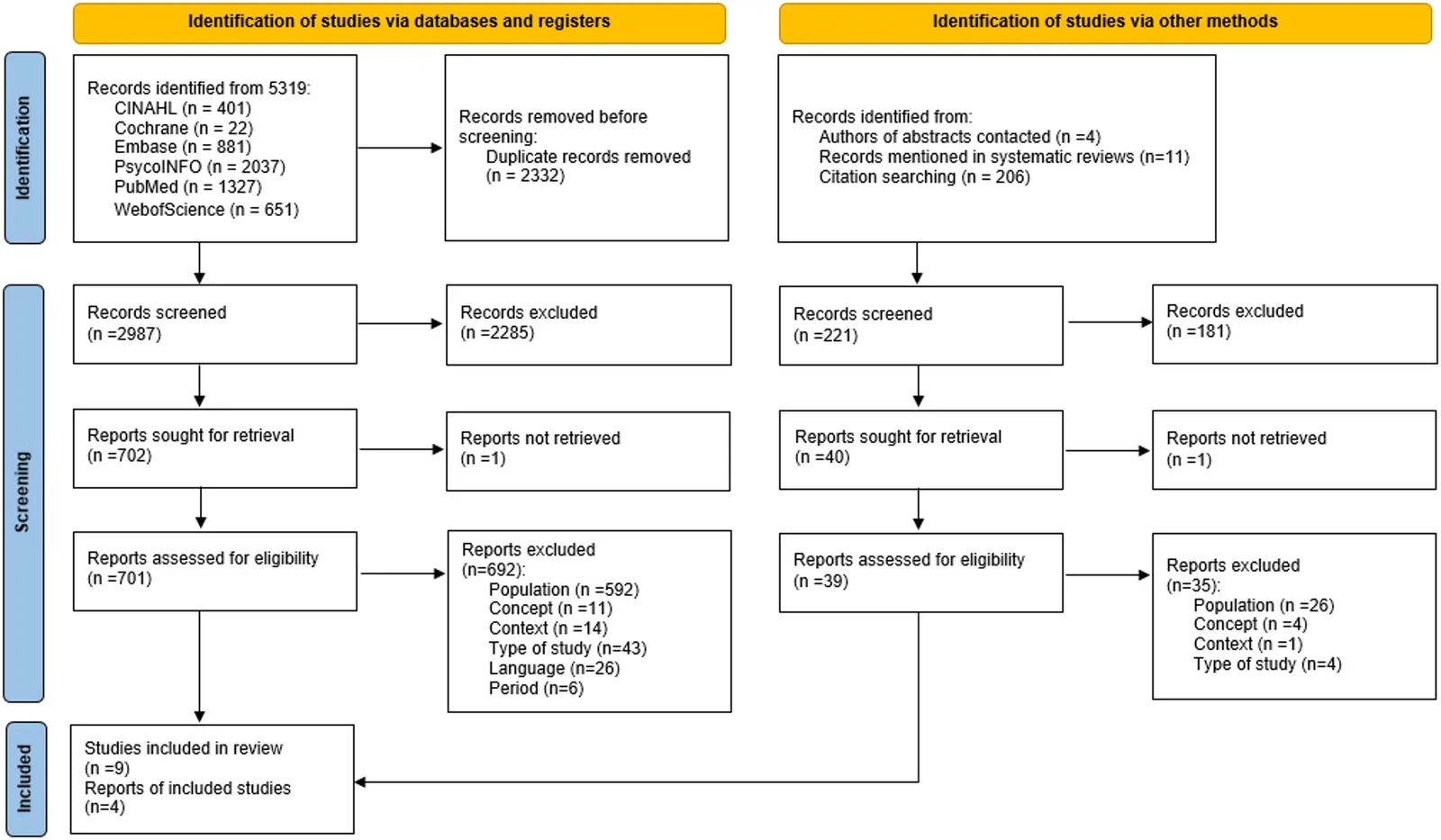
PRISMA flow diagram. *From*: Page MJ, McKenzie JE, Bossuyt PM, Boutron I, Hoffmann TC, Mulrow CD, et al. The PRISMA 2020 statement: an updated guideline for reporting systematic reviews. BMJ 2021;372:n71. https://doi.org/10.1136/bmj.n71

A total of 5540 records were identified (Fig. 1). After duplicated records were removed, 3208 were reviewed by title and abstract. Of them, 740 records were reviewed in full text. Finally, 13 records were included in this study.

### Records Included

All records were published between 2000 and 2021, three in the first decade of the period (2000–2010) [28–30], one between 2011 and 2014 [31], and nine after 2015 [32–40]. Studies were conducted in Spain (*n* = 7) [28–30, 32, 36, 37, 39], France (*n* = 4) [31, 33, 38, 40] and Germany (*n* = 2) [34, 35]. Most (*n* = 9) were cross-sectional [28–30, 32, 34–36, 39, 40], three were case reports [31, 33, 37], and one was qualitative in design [38]. Seven studies included only migrants from NA [28–31, 33, 37, 38], and six also included migrants from other origins [32, 34–36, 39, 40]. All studies covered aspects related to health status, two of them aspects related to the provision of health service [31, 38], and no study described the use of health services. The Table 2 presents the characteristics of all included records.

**Table 2:** Baseline characteristics of included records

Ref	Author and publication year	Study design	Country	Participants country of origin	Participants age range	Number of female participants	Participants eligibility	Sample size	Study period	Health condition explored	Service provision explored
[28]	Oliván Gonzalvo 2000	Cross-sectional	Spain	Morocco Algeria	13 to 17	None	Unaccompanied minors in residential protection centers	49	1997–1999	Health status, oral health, nutrition	None
[29]	Rivas Clemente et al. 2000	Cross-sectional	Spain	Maghreb	14 to 24	NS*	Included in the individual card system	12	1996–1998	Infection diseases	None
[30]	Oliván Gonzalvo 2004	Cross-sectional	Spain	Morocco Algeria	12 to 17	None	Unaccompanied minors in residential protection centers	120	1997–2003	Health status, infectious diseases, oral health, nutrition	None
[31]	Harscoet et al. 2013	Case report	France	Algeria	16	1	NA**	1	NS*	Mental health	Cognitive and behavioural therapy, addressing cultural elements
[32]	Collazos Sánchez et al. 2015	Cross-sectional	Spain	Morocco Algeria	12 to 17	None	Unaccompanied minors in residential protection centers	98	NS*	Mental health	None
[33]	Ludot et al. 2015	Case report	France	Tunisia	18	None	NA**	1	NA**	Mental health	None
[34]	Marquardt et al. 2016	Cross-sectional	Germany	Morocco Algeria Tunisia	12 to 18	NS*	Unaccompanied minors seeking for international refugee protection	13	2011–2014	Health status, infectious diseases, mental health, oral health	None
[35]	MaaBen et al. 2017	Cross-sectional	Germany	Morocco Algeria	13 to 18	None	Unaccompanied minors in reception centers	4	2014–2015	Infectious diseases	None
[36]	De la Parte Serna et al. 2019	Cross-sectional	Spain	Morocco Algeria	NS* (mean 15.9; SD 1.5)	None	Unaccompanied minors in residential protection centers	528	2005–2018	Oral health	None
[37]	Tolosa Fortuny et al. 2019	Case study	Spain	Morocco	10 and 13	None	NA**	2	2016–2017	Infectious diseases	None
[38]	Radjack et al. 2020	Case study	France	Morocco	17	None	NA**	1	Unknown	Mental health	Transcultural approach
[39]	Oliván Gonzalvo 2021	Cross-sectional	Spain	Morocco Algeria	13 to 17	None	Unaccompanied minors in residential protection centers	534	2005–2019	Health status, infectious diseases, mental health, nutrition	None
[40]	Bergevin et al. 2021	Cross-sectional	France	Morocco Algeria Tunisia	NS*	NS*	Unaccompanied minors	14	2017–2018	Health status, infectious diseases	None

*NS: Not specified: **NA: Not applicable

### Studied Population

In total, the 13 records reported about 1374 migrants from NA, with a range from one to 534 per record. It is worth noticing, however, that twice, two studies seem to refer to the same participants [28, 30, 36, 38]. If this is the case, the actual number of migrants from NA included is 797 as 577 likely participated in more than one study.

Six studies included participants from both Morocco and Algeria [28, 30, 32, 35, 36, 39]. Overall, study participants were mainly from Morocco (1150 in 12 studies) and Algeria (178 in 10 studies). There was only one participant from Tunisia, a case report [33]. Thirty-nine participants in three studies were described as being from NA, with no mention of their country of origin [29, 34, 40].

It was not possible to consistently assess a range or an average age of participants. Ten studies covered an age range of 12 to 18 years old [28, 30–32, 34–36, 38–40]. One of the case reports included a patient who was 10 years old [37]. Only two studies included participants of 18 to 24 years old [29, 33].

Nine studies included only males [28, 30, 32, 33, 35–39] and three both male and female migrants [29, 34, 40], although in relatively low proportion of the later (11% to 23.5%). These three studies included also migrants from countries other than NA, or from age groups older than 24 years old, and did not provide information about the number of female participants disaggregated by age and country of origin. One case report described the case of a female patient [31]. Therefore, it was not possible to precisely assess the proportion of female participants in the studies included in our review.

Being an unaccompanied minor was an inclusion criterion for eight studies [28, 30, 32, 34–36, 39, 40] and two case reports focused on an unaccompanied minor and unaccompanied young adult [31, 33].

### Health Status

All studies explored aspects related to the health status of participants. Five explored aspects related to general health, seven to infectious diseases, six to mental health including substance use, four to oral health, and three to nutritional status. Main results are presented in Table 3.

**Table 3:** Key findings in health status reported by included records

Domains	Reported prevalence	References
General health	At least one health problem: from 50% to 77% More than one health problem: from 15% to 44% Gastritis symptoms: 38% Others: dental caries, ferritin and iron deficiency, wounds and injuries	[28, 30, 39] [28, 30] [34] [30]
Infectious diseases	Infectious diseases: 53.8% Latent TB: from 6% to 27% Active TB: from 0% to 1.2% Skin infections: from 11.6% to 17.6% Intestinal parasitosis: from 0% to 4.6% Others: mention of leishmaniasis	[34] [30, 39, 40] [30, 39, 40] [30] [30, 35, 39, 40] [37]
Mental health	Mental health disorders: 7.7% PTSD: 14% Substance-related disorders: 13% Others: mention of complex PSTD, ADHD and social anxiety	[34] [39] [32] [31–33]
Oral health	Oral health problems: from 30.8% to 32.5% Caries: from 27.5% to 35.3%	[28, 34] [30, 36]
Nutritional health	Iron deficiency: from 5.9% to 27.9% Mild malnutrition: 12.5% Overweight: 4.3% Obesity: 0.4%	[28, 39] [28] [39] [39]
Others	Mention of physical trauma and injuries	[38]

### General Health

The prevalence of any health problem was provided in three studies [28, 30, 39] which investigated the health status of unaccompanied minors in residential protection centres in Spain. The reported prevalence of suffering at least one health problem varied depending on the study, amounting to 50% [28], 71% [39], and 73% and 77% among those from Algeria and Morocco respectively [30]. The presence of more than one health problem ranged from 15% [28], to 35% among migrants from Algeria and 44% among those from Morocco [30].

The most prevalent health conditions reported were, by order of frequency, dental caries, iron deficiency with or without anaemia, wounds and injuries, infectious or parasitic dermatitis, impaired visual acuity and imported infectious and parasitic diseases [30].

A study conducted in Germany explored the health status of unaccompanied minors seeking asylum and reported the results of their initial medical check-ups [34]. This study found a prevalence of gastritis symptoms in 38% of NA migrants, which was lower than among migrants from Sub-Saharan Africa (60%), but higher than among those from South Asia and West Asia (29% and 7% respectively).

### Infectious Diseases

The overall prevalence of infectious diseases was reported in one study, as 53.8% among NA, lower than among Sub-Saharan migrants, but higher that among migrants from other regions [34].

Latent and active tuberculosis (TB) were reported in three studies exploring health status of newly arrived unaccompanied minors. The reported prevalence of latent TB varied depending on the study between 6% [30], 8.2% [39] and 27% [40]. Similarly, the prevalence of active TB varied between 0% [40], 0.8% [39] and 1.2% [30]. Another study explored the prevalence of tuberculosis infection among NA migrants living in a basic health zone in Spain [29]. Authors found that 33.3% of 14 to 24 years old migrants had a Mantoux test of more than 15 mm, 50% of more than 10 mm and 58,3% of more than 5 mm. They suggested that their poor living conditions in the receiving country could partly explain this high prevalence.

Leishmaniasis was described in one study reporting three cases within the age group defined in this review [37]. Cases originated from three families having visited the same area in Morocco. All were considered as imported cases. The authors mentioned difficulties in the diagnosis of cases, with false negative results of histopathology, eventually confirmed by PCR. Difficulties in case management were also described, notably in relation to acceptance of health services by the parents.

Skin infections were described in one study [30], reporting a prevalence of 11.6% among Moroccan and 17.6% among Algerian migrants. Other infections, including past and present Hepatitis B virus (HBV) infection, schistosomiasis, malaria, intestinal parasitosis, HIV, and syphilis, were explored in four studies assessing the health status of unaccompanied minors at the time of arrival [30, 35, 39, 40]. The prevalence of intestinal parasitosis was reported as 0%, 0.9% and 4.6% in the different studies. No study reported active infections with malaria, schistosomiasis, HIV, or syphilis.

### Mental Health and Substance Use

A study conducted among unaccompanied asylum seeker minors indicated a prevalence of mental health disorders (including depression and Post Traumatic Stress Disorder, PTSD) of 7.7% in those from NA, which was lower than in minors from sub-Saharan Africa (10%) and West Asia (20%) [34].

Psychological trauma was mentioned in three studies. One of them described a prevalence of PTSD of 14% in unaccompanied minors originating from NA [39]. Two case reports described the clinical cases of unaccompanied minor and a young adult suffering from PTSD [33, 38]. The authors mentioned the presence of “multiple traumas still encysted and active” and the history of suicide attempt, behavioural problems and depressive symptoms, and discussed the concept of “complex PTSD” to describe personality changes secondary to a history of trauma [33].

Two studies mentioned substance use. The first study explored this condition among unaccompanied minors from NA hosted in minors’ centres [32]. 13% of the study participants fulfilled the criteria of dependence and abuse of substance others than alcohol, based on Mini International Neuropsychiatric Interview for Children and Adolescents (MINI-KID). Researchers also found that 53% had used more than one substance in the last month. The median age of first use was 13.9 and 15.7 years for solvents and cocaine respectively. The study described also a discrepancy between self-reported consumption and toxicologic hair analysis of benzodiazepines use, with the last methods indicating a prevalence of 22%. Lastly, the study found a correlation between the amount of cannabis and solvent consumption and having suffered some type of physical abuse in adolescence. In a case report describing an unaccompanied minor, the authors mentioned an history of addiction to different substances (including glue, silicium, solvents, cannabis and psychotropic) as part of the case presentation [38].

Other mental health conditions reported were Attention Deficit Hyperactivity Disorder (ADHD) and social anxiety. ADHD prevalence was reported as 28% with a global T-score above 70, notably due to hyperactivity values, using Conners scale [32]. Social anxiety was mentioned in a case report describing the therapy offered to a minor originally from Algeria living in France with her parents [31].

### Oral Health

The prevalence of oral health problems was reported in two studies conducted among newly arrived unaccompanied minors. The reported prevalence ranged from 30.8% [34], to 32.5% [28].

Similar values were observed about the more specific presence of caries in two other studies. The reported prevalence of dental caries was consistently above 27.46% with values at 33.7% and 35.3% in various studies and countries of origin [30, 36]. Indeed dental caries was described as the second most common health problem among unaccompanied minors from NA, with a prevalence significantly higher than that of migrants from Sub-Saharan Africa [36].

### Nutritional Status

Nutritional anthropometric parameters were explored in two studies. While one study did not find a statistically significant difference between adolescent migrants from NA and the national standards of the receiving country [28], another revealed intra-regional variation within the NA group itself [30]. Specifically Moroccan migrants demonstrated significantly lower measurements in weight, brachial circumference and subscapular skinfold values compared to Algerian peers.

Nutritional index was reported in one study, describing its mean value within the normal range [28]. However, an individualized analysis of this index showed a mild form of acute malnutrition in 12.5% of the participants from NA.

Another study, found a prevalence of obesity and overweight of 0.4% and 4.3% respectively [39] and concluded that unaccompanied minors from NA were significantly less frequently overweight that those from other origins.

Iron deficiency was also explored in three studies. One study reported a prevalence of iron deficiency with or without anaemia of 5.9% for unaccompanied migrants from Algeria and 8.1% for those from Morocco [28]. The study found also a prevalence of ferritin isolated deficiency (latent iron deficiency) of 2.9% among Algerians and 18.6% among Moroccans. This study indicated indeed that iron deficiency (isolated ferritin deficiency or iron deficiency with or without anaemia) was the second most prevalence health disorder within this group. When compared with migrants from other regions, the prevalence of iron deficiency among unaccompanied minors from NA was 27.9%, significantly higher than among participants from Sub-Saharan Africa [39]. The reported prevalence of iron deficiency anaemia ranged from 2.5% to 7.1% [28, 39].

### Other Conditions

Physical trauma and injuries are mentioned in one study, describing an unaccompanied minor with an instance of two legs fractured in the context of a violent trauma [38].

### Health Services Provision and Use

Only two articles explored aspects related to health services provision and both focused on provision of mental health therapy [31, 38]. They described a cognitive behavioural therapy targeting a person with social anxiety diagnosis [31] and a transcultural therapy addressing the psychological distress of unaccompanied migrants in a hospital [38].

Both studies mentioned the importance of taking into account the cultural elements in the analysis and treatment of these young migrants and one of them [38] highlighted the benefits of strengthening the cross-cultural competencies of health professionals, social workers in this case.

This last study [38] discussed four main dimensions of critical importance when delivering care to wandering unaccompanied minors: the understanding of the journey and goals of the foreign minor by considering the political and historical counter-transferential aspects, the elaboration on the traumatic elements to transform their effects in therapeutic levers, the creation of a bond of trust, and the commitment of those involved in providing care.

No study specifically reported about health services access.

## Discussion

To our knowledge, this scoping review is the first comprehensive analysis of the literature about the health status, access to services and health services provision of North African adolescent and young adult migrants in Europe. Population studied were mainly composed of migrants from Morocco and Algeria, unaccompanied minors and males between 12 and 18 years old. Evidence was mostly generated by cross-sectional studies exploring their health status and giving special attention to infectious diseases, mental health and, to a lesser extent, general health. The attention given to health service provision was limited, and most authors stressed the importance of considering cultural aspects. This scoping review did not identify any study exploring access to health services.

Our review highlights that evidence on this topic is scarce in quantity and of limited methodological quality. The number of studies conducted and the number of individual participants, overall and within each study, are small especially when contrasted with the size of the study population. While exact numbers about the North African adolescent and young adults first generation migrants living in Europe do not exist, available statistics indicate that the number of NA migrants living in Europe is possibly as large as 5 million [2]. Similarly, information about new arrivals in Europe indicate that NA countries were among the ten top countries of origin from 2017 to 2024 [41]. Evidence provided is also questionable in terms of quality as most of the studies conducted on this topic were cross-sectional or case studies. These gaps stress the need of additional studies focused on adolescent and young adult first generation migrants from NA, to inform health policies and service delivery adaptation.

This review could not precisely assess the proportion of female participants in the studies included, although information available suggested it was very low. The absence of official statistics on the number of adolescent and young female migrants from NA in Europe prevents from drawing comparisons and interpretations with the results of the studies included in this review. Actually, migration figures in Europe indicate that an important percentage of migrants are women, including those from NA [42]. In the same line, scholars described the growing phenomenon of young females from Morocco traveling by themselves to Europe and referred to them as female “harraga” in reference to their male counterparts [43]. A study conducted in France, exploring the situation of first and second generation young female migrants from NA, also stressed knowledge gaps about women migrants from NA and described them as “forgotten” from public discourses in comparison to males [44]. The author suggested that migrant women may express their specific discomfort in a less spectacular way than men, making it somehow less visible or even invisible. Similarly, a study conducted in Italy highlighted the limited knowledge on mental health in NA migrants in Europe, particularly among women [45]. Both studies described specific health needs of NA women such as depression, substance use, denial of pregnancy, higher scores of endogenous stress and discomfort, and lower scores of well-being and quality of life than women born in Europe. Although their conclusions should be cautiously compared to our study population, as the first of these two studies referred to a majority of second-generation migrants and the second to a larger age group which included persons older than 24 years old, they both highlight knowledge gaps for female NA migrants and describe the existence of specific health needs. Overall, these studies and ours point to the underrepresentation of North African female migrants in recent research and what seems to be a clear blind spot about their potential specific health status. More broadly, the sex and gender gap in medical research has been well-documented, with demonstrable negative implications for female health outcomes across both the general population and migrant groups [46–49]. Increasingly, the health research community has acknowledged the urgency of addressing this disparity – not only in scientific inquiry but also in the design and delivery of health services- in order to promote more equitable health outcomes [50–52]. The results of our review align with these concerns and highlight the pressing need to better understand the health status of adolescent and young women migrants from NA, including first generation. Such evidence is essential to inform the adaptation of health services that can more effectively respond to the specific needs of this population.

The results of our review showed that most of the knowledge about adolescents and young migrants from NA is focused on those who are less than 18 years old, with limited attention to those who are older. A study exploring the experiences of unaccompanied young migrants after leaving childcare and their transition to adulthood also identified important evidence gaps for this age-group [53]. Another report analysing the support offered to such migrants by governments in different European countries stressed the specific vulnerability during this transition period, and highlighted the specific risks that this moment represents for their mental health [54]. This period is developmentally distinct and has a strong influence on the long-term life trajectories of individuals. However, a report on the health of young adults stressed how little is known on this topic and suggested that this lack of evidence may be the result of the combination of children and adults in most statistical reporting and research design [55]. In addition, some reports describe that many unaccompanied minor migrants become undocumented after turning 18 [56]. As a result, and because they fall outside of foster services attention, documenting their health status and interactions with health services represents additional methodological challenges. This may, at least in part, explain this lack of knowledge about young adult migrants from NA. Facing this challenge using the adequate methodological designs is indispensable to address this knowledge gap.

Our study shows that oral health and iron deficiency are the most prevalent somatic health conditions among North African adolescent and young adult migrants identified in the literature. This is in line with results from a narrative review about refugees and undocumented children in Europe, which found that oral health and nutritional deficiencies were more common within children on the move, oral health being the most prevalent health problem [57]. This scoping review shows that tuberculosis and leishmaniasis are the infectious diseases most often mentioned among adolescent and young adult migrants from NA. Both are also described as of important concern in terms of prevalence in countries of origin and among migrants from these regions, in a review about infectious diseases in North African migrants in Europe [58]. Lastly, our study suggests that mental health, including PTSD and substance use, is an important health problem among adolescent and young adult migrants in NA. The narrative review mentioned above presented mental health as the main risk for children on the move and discussed mental health as the most challenging for health service provision [57]. This is also in line with a UNICEF report indicating an elevated risk of mental health problems in children and youth migrants due to cumulated adverse-life events along their migration continuum [59]. More concretely, high prevalence of psychological trauma exposure, and substance use have been described in forced migrants and adolescents refugees, as well as the correlation between the two [60, 61]. This points at the need to pay special research attention and possibly devoted health care services to oral health, nutritional deficit, tuberculosis, leishmaniasis, PTSD and substance use.

The results of our study revealed that most of the health knowledge on adolescent and young adult migrants from NA is focused on assessing their health status, with very limited to not existing studies focused on healthcare programs or use of health services respectively. As outlined by Levesque, both the demand side and supply side dimensions are essential when evaluating the accessibility of health services [62]. Understanding accessibility across the full pathway of service utilisation is particularly critical for vulnerable populations. The knowledge gap identified in our review, contrasts with a relatively larger knowledge on this topic for minor and young adults migrants in general [63–65]. This discrepancy may reflect specific difficulties faced by NA-origin adolescents and young adult in accessing health services, particularly regarding continuity of care. These challenges likely contribute to their underrepresentation in service utilisation studies and underscore the need for focused, population-specific investigation. While some of the knowledge from minor and young adult migrants from other origins may be applicable to those from NA, the extent of this applicability is debatable. Therefore, the accessibility of existing health services for this population requires more rigorous and targeted investigation.

Our study has some limitation that are worth mentioning. First, only literature in English, French and Spanish was included. It is possible that additional information could have been identified in other languages, such as Arabic or any European language. Second, for practical reasons, we opted for not conducting a search in general, governmental or institutional websites. It may have provided additional insights. This decision was partly compensated, by favouring sensitivity in the design of the search strategy and the decision to include non-indexed reports. Last, in the absence of internationally agreed age range for adolescence and young adulthood, we defined them as individuals from 10 until 24 years old. This may have limited our ability to identify issues prevalent beyond these limits. Overall, these limitations do not invalidate our main findings and stress indeed the scarcity and the relatively poor quality of the literature on this topic.

## Conclusion

This study is the first comprehensive analysis of the literature about the health status, access to services and health service provision of North African adolescent and young adult migrants in Europe. It revealed an overall limited knowledge on this topic and major gaps about females and young adults, as well as in healthcare programs and use of services. These knowledge gaps result and contribute to perpetuate inadequate policies and ineffective health interventions. As a result, existing inequalities for this group are exacerbated. As oral health, iron deficiency, leishmaniasis, tuberculosis, mental health and substance use are the most often quoted health conditions, there may be prioritized for research and receive specific attention in health care. Further research is needed to enhance a comprehensive, complex and reflective understanding of this population, including their health status, migration route, personal history and risk factors. Research design should emphasize gender perspective, include child and youth-centeredness, and use methodologies suitable for hard-to-reach and under the radar “invisible” populations. Their high mobility within European countries calls for international collaboration and cross-border research.

## Supplementary Information

Below is the link to the electronic supplementary material.

Supplementary Material 1

Supplementary Material 2

## Data Availability

No datasets were generated or analysed during the current study.
