# Correction: Health Status of North African Adolescent and Young Adult Migrants in Europe: a Scoping Review

**DOI:** 10.1007/s10903-025-01833-4

**Published:** 2025-12-06

**Authors:** Eva P. Rocillo Aréchaga, Barbara Broers, Catherine Chamay Weber, Delphine Courvoisier, Lloyd Orphée Rigumye, Melanie Pinon, Yves-Laurent Jackson

**Affiliations:** 1 Division of Primary Care Medicine, Geneva University Hospitals (HUG), Geneva, Switzerland; 2 Institute of Global Health Faculty of Medicine, University of Geneva (UNIGE), Geneva, Switzerland; 3 Department of Community Health and Medicine Faculty of Medicine, University of Geneva (UNIGE), Geneva, Switzerland; 4 Division of General Paediatrics Department of Women, Child and Adolescent Adolescent Health Unit, Geneva University Hospitals (HUG), Geneva, Switzerland; 5 Division of Quality of Care, Geneva University Hospitals (HUG), Geneva, Switzerland; 6 Department of Medicine Faculty of Medicine, University of Geneva (UNIGE), Geneva, Switzerland; 7 University of Applied Sciences and Arts Western Switzerland School of Health Sciences (HES-SO), Geneva, Switzerland

Correction to: Journal of Immigrant and Minority Health.

https://doi.org/10.1007/s10903-025-01821-8.

In this article, the word “Title” prefix the actual article title and was incorrectly published as ' Tittle: Health Status of North African Adolescent and Young Adult Migrants in Europe: a Scoping Review ' but should have been ‘Health Status of North African Adolescent and Young Adult Migrants in Europe: a Scoping Review ‘.

The original article has been corrected.

