# Supplementary material for: Health Status of North African Adolescent and Young Adult Migrants in Europe: a Scoping Review
Source: J Immigr Minor Health. 2025 Nov 15;28(5):1233–44. doi: 10.1007/s10903-025-01821-8 (PMC13612637; doi:10.1007/s10903-025-01821-8)
Supplement: Supplementary file 1 [file 10903_2025_1821_MOESM1_ESM.docx]

**Annex 1.** Search strategy

|  | Query PubMed |
| --- | --- |
|  |  |
| #1 | ("Adolescent"[MeSH Terms] OR "Adult"[MeSH Terms:noexp] OR "Young adult"[MeSH Terms] OR "Child"[MeSH Terms] OR "adolescence"[Title/Abstract] OR "adolescent*"[Title/Abstract] OR "adult*"[Title/Abstract] OR "child*"[Title/Abstract] OR "childhood"[Title/Abstract] OR "minor*"[Title/Abstract] OR "prime adult*"[Title/Abstract] OR "teen*"[Title/Abstract] OR "young*"[Title/Abstract] OR "youth*"[Title/Abstract]) AND (1990/1/1:3000/12/12[pdat]) |
| #2 | ("africa, northern"[MeSH Terms:noexp] OR "Algeria"[MeSH Terms] OR "Morocco"[MeSH Terms] OR "Tunisia"[MeSH Terms] OR "Libya"[MeSH Terms] OR "algeria*"[Title/Abstract] OR "Libia"[Title/Abstract] OR "Libya"[Title/Abstract] OR "lybia*"[Title/Abstract] OR "maghreb*"[Title/Abstract] OR "Maghrib"[Title/Abstract] OR "Moroccan"[Title/Abstract] OR "Morocco"[Title/Abstract] OR "north africa*"[Title/Abstract] OR "northern africa*"[Title/Abstract] OR "Sahara"[Title/Abstract] OR "tunisia*"[Title/Abstract]) AND (1990/1/1:3000/12/12[pdat]) |
| #3 | ("Transients and Migrants"[MeSH Terms] OR "Emigrants and Immigrants"[MeSH Terms] OR "Refugees"[MeSH Terms] OR "alien*"[Title/Abstract] OR "asylum seeker*"[Title/Abstract] OR "emigrant*"[Title/Abstract] OR "foreigner*"[Title/Abstract] OR "immigrant*"[Title/Abstract] OR "migrant*"[Title/Abstract] OR "migration"[Title/Abstract] OR "inmigration"[Title/Abstract] OR "nomad*"[Title/Abstract] OR "refugee*"[Title/Abstract] OR "squatter*"[Title/Abstract] OR "transient*"[Title/Abstract]) AND (1990/1/1:3000/12/12[pdat]) |
| #4 | ("Health"[MeSH Terms] OR "Health Status"[MeSH Terms] OR "Health Status Indicators"[MeSH Terms] OR "Risk Factors"[MeSH Terms] OR "Morbidity"[MeSH Terms] OR "Mortality"[MeSH Terms] OR "Mental Health"[MeSH Terms] OR "Substance-Related Disorders"[MeSH Terms] OR "clinical state"[Title/Abstract] OR "drug abuse"[Title/Abstract] OR "drug addict"[Title/Abstract] OR "drug addiction"[Title/Abstract] OR "drug dependenc*"[Title/Abstract] OR "drug use disorder*"[Title/Abstract] OR "health determinant*"[Title/Abstract] OR "health disparit*"[Title/Abstract] OR "health inequit*"[Title/Abstract] OR "health level*"[Title/Abstract] OR "heath stat*"[Title/Abstract] OR "level of health"[Title/Abstract] OR "mental condition"[Title/Abstract] OR "mental disorder*"[Title/Abstract] OR "Mental Health"[Title/Abstract] OR "mental status"[Title/Abstract] OR "morbidities"[Title/Abstract] OR "mortalities"[Title/Abstract] OR "physical health"[Title/Abstract] OR "population at risk"[Title/Abstract] OR "psychic health"[Title/Abstract] OR "psychosis"[Title/Abstract] OR "psychotic disorder*"[Title/Abstract] OR "risk factor*"[Title/Abstract] OR "subclinical psychotic symptom*"[Title/Abstract] OR "substance abuse*"[Title/Abstract] OR "substance dependenc*"[Title/Abstract] OR "substance use"[Title/Abstract] OR "toxicomania*"[Title/Abstract]) AND (1990/1/1:3000/12/12[pdat]) |
| #5 | ("Health Services"[MeSH Terms] OR "Health Services Needs and Demand"[MeSH Terms] OR "Health Services Accessibility"[MeSH Terms] OR "Patient Acceptance of Health Care"[MeSH Terms] OR "Mental Health Services"[MeSH Terms] OR "Health Education"[MeSH Terms] OR "acceptability"[Title/Abstract] OR "access to care"[Title/Abstract] OR "access to therapy"[Title/Abstract] OR "access to treatment"[Title/Abstract] OR "accessibility"[Title/Abstract] OR "attitude*"[Title/Abstract] OR "barrier*"[Title/Abstract] OR "health care"[Title/Abstract] OR "healthcare"[Title/Abstract] OR "Health Education"[Title/Abstract] OR "health information"[Title/Abstract] OR "health prevention"[Title/Abstract] OR "health promotion"[Title/Abstract] OR "health promotion"[Title/Abstract] OR "health service*"[Title/Abstract] OR "health utilization"[Title/Abstract] OR "health seeking behaviour"[Title/Abstract] OR "preventive program*"[Title/Abstract] OR "preventive service*"[Title/Abstract] OR "psychiatric service*"[Title/Abstract] OR "suburban health service"[Title/Abstract] OR "uptake"[Title/Abstract] OR "use"[Title/Abstract] OR "utilization"[Title/Abstract]) AND (1990/1/1:3000/12/12[pdat]) |
| #6 | ("prisoners"[MeSH Terms:noexp] OR "prisons"[MeSH Terms] OR "convicted offender*"[Title/Abstract] OR "custodial setting*"[Title/Abstract] OR "detain*"[Title/Abstract] OR "convict*"[Title/Abstract] OR "convicted felon"[Title/Abstract] OR "correctional centre*"[Title/Abstract] OR "correctional setting*"[Title/Abstract] OR "criminal conviction"[Title/Abstract] OR "detention*"[Title/Abstract] OR "imprison*"[Title/Abstract] OR "incarcerat*"[Title/Abstract] OR "inmate*"[Title/Abstract] OR "jail*"[Title/Abstract] OR "penal institut*"[Title/Abstract] OR "prison"[Title/Abstract] OR "remand centre*"[Title/Abstract] OR "incarcerated offender*"[Title/Abstract] OR "incarcerated population"[Title/Abstract] OR "penitentiar*"[Title/Abstract] OR "prisoner*"[Title/Abstract]) AND (1990/1/1:3000/12/12[pdat]) |
|  | #1 AND #2 AND #3 AND #4 |
|  | #1 AND #2 AND #3 AND #5 |
|  | #1 AND #2 AND #3 AND #6 |
|  | #2 AND #3 AND #4 |
|  | #2 AND #3 AND #5 |
|  | #2 AND #3 AND #6 |
|  | #2 AND #3 |
|  | #1 AND #2 AND #3 |
