# Supplementary material for: Health Status of North African Adolescent and Young Adult Migrants in Europe: a Scoping Review
Source: J Immigr Minor Health. 2025 Nov 15;28(5):1233–44. doi: 10.1007/s10903-025-01821-8 (PMC13612637; doi:10.1007/s10903-025-01821-8)
Supplement: Supplementary file 2 [file 10903_2025_1821_MOESM2_ESM.docx]

**Annex 2.** Data abstraction form

| **Data extraction form** | |
| --- | --- |
| I. Study ID information | |
| Study ID | First Name-Publication Year |
| First Author | Name |
| Was author contacted? | 1 – Yes 2 – No If yes, dates(s) |
| If yes, author response? |  |
| Study data | 1 – Peer review article 2 – Non-indexed report 3 – Ongoing  4 – Other |
| Title |  |
| Year (of publication) | YYYY |
| Year study start date | YYYY or 2 – Not reported |
| Language | 1 – English 2 – Spanish  3 – French |
| II. Study details | |
| Country (ies) where study was conducted | List all |
| Study objective | Describe or 2 -Unknown/Not reported |
| Study design | 1 – Randomized controlled trial  2 – Non-randomized controlled trial 3 – Cross-sectional 4 – Cohort 5 – Case-control  6 – Case study  7 – Mixed-methods  8 - Other, specify 9 – Could not tell  If other, describe |
| Participant allocation/selection | 1 - Consecutive  2 - Random  3 - Convenience  4 – Purposive  5 – Other  9 - Unknown/Not reported |
| Direction of study data collection | 1 – Prospective 2 – Retrospective  3 - Cross-sectional 9 – Unknown/Not reported |
| Dates of data collection | 1 - From MM/YY to MM/YY  2 - Other, specify 9 – Could not tell |
| Duration of follow up (months) |  |
| III. Participants and setting characteristics | |
| Country of origin | 0 – Morocco 1 – Algeria 2 – Tunisia  3 – Libya  4 – Other. Describe 9 – Unknown/Not reported |
| Age (mean, as described by authors) | 1 – XX 2 – Other. Describe 9 – Unknown/Not reported |
| Sex, female: ___% | 1 – XX 2 – Other. Describe 9 – Unknown/Not reported |
| Only NA migrants included in the study | 1 – Yes 2 – No |
| Comparison group | 0 – No comparison group  1 – Yes. Describe  2 – Others. Describe  9 – Unknown/Not reported |
| Number of participants recruited (add) |  |
| Participants inclusion criteria | 1 - Country of origin. Describe  2 - Age. Describe  3 - Legal status. Describe  4 - Medical condition. Describe  5 - Using health service. Describe  6 - Other. Describe:  9 - Not reported |
| Participants exclusion criteria | 1 - Country of origin. Describe  2 - Age. Describe  3 – Gender. Describe  4 – Legal status. Describe  5 - Medical condition. Describe  6 - Other. Describe  9 - Not reported |
| Setting | 1 – Hospital services  2 - Primary health services  3 – Community health services  4 – NGOs  5 – Schools  6 – Protection center  7 – Prison setting 8 – Others. Describe:  9 – Unknown/Not reported |
| VI. Concept explored in the study | |
| Main concept explored | 1 – Health status  2 – Use of health service  3– Accessibility of health service  4 - Health service offered  5 - Others. Describe:  9 – Unknown/Not reported |
| Medical condition explored | 1 – General health  2 – Infectious diseases  3 – Mental health  4 - Substance use  5 - Nutritional status  6 - Oral health  7 - NCD  8 - Mortality  9 - Others. Describe:  0 – Unknown/Not reported |
| Health service type (use) | 1 -Hospital  2 - Primary health services  3 - Community health/GP  4 - NGOs  5 - Outreach health  6- -Other. Describe  9 - Unknown/Not reported |
| Accessibility aspect explored | 0 - Not relevant  1 - Describe |
| Health service type (offered) | 0 - Not relevant  1 -Hospital  2 - Primary health services  3 - Community health/GP  4 - NGOs  5 - Outreach health  6- -Other. Describe  9 - Unknown/Not reported |
| Health provider characteristic | 0 - Not relevant  1 - General doctor  2 - Specialized doctor  3 - Nurse  4 - Physiotherapist  5 - Psychologist  6 - Other. Describe  9 - Unknown/Not reported |
| Policy explored | 0 - Not relevant  1 - Describe |
| V. Outcomes | |
| Outcomes evaluated  (describe each, add) | 0 - Not relevant  1 - Incidence  2 - Prevalence  3 - Service uptake  4 - Service provided  5 - Others. Describe |
| Recommendations (add) | Describe |
